# Psychometric evaluation of the Brief Resilient Coping Scale (BRCS) over the course of the pandemic in a large German general population sample

**DOI:** 10.1371/journal.pone.0309587

**Published:** 2024-08-27

**Authors:** Julia Petersen, Elmar Brähler, Nora Hettich-Damm, Markus Schepers, Jochem König, Karl Lackner, Norbert Pfeiffer, Manfred E. Beutel

**Affiliations:** 1 Department of Psychosomatic Medicine and Psychotherapy, University Medical Center of the Johannes Gutenberg-University Mainz, Mainz, Germany; 2 Department of Medical Psychology and Medical Sociology, Medical Faculty, University of Leipzig, Leipzig, Germany; 3 Institute of Medical Biostatistics, Epidemiology and Informatics, University Medical Center of the Johannes Gutenberg-University Mainz, Mainz, Germany; 4 Institute of Clinical Chemistry and Laboratory Medicine, University Medical Center of the Johannes Gutenberg-University Mainz, Mainz, Germany; 5 Department of Ophthalmology, University Medical Center of the Johannes Gutenberg-University Mainz, Mainz, Germany; Johns Hopkins Medicine, UNITED STATES OF AMERICA

## Abstract

**Background:**

The Brief Resilience Coping Scale (BRCS) is a brief instrument suitable for epidemiological studies. The aims of this paper were to analyze changes in BRCS depending on time, sex, age group, relationship status, as well as risk of poverty, to test the psychometric properties including test-retest reliability and measurement invariance, and to determine associations with psychosocial stress, depressiveness, anxiety, social support, as well as subjective mental and physical health. As the data from this study was collected during the pandemic, an additional sensitivity analysis was performed with pre-pandemic data.

**Methods:**

A longitudinal study of resilience and distress in a large-sized community sample was performed at one pre-pandemic (T0) and three pandemic time points (T1-3). Resilient coping was assessed by the 4-Item short form of the BRCS, distress by the PHQ-9 and GAD-2.

**Results:**

BRCS decreased between the first and the second and increased at the third pandemic time point. The scale had a good internal consistency. Test-retest correlation coefficients ranged from 0.527 to 0.589. Higher resilient coping was found in younger participants, participants not at-risk-of-poverty and in males. Stability was higher in those with a partner, and at-risk-of-poverty. Significant negative associations with psychosocial stress, loneliness, depressiveness, anxiety, social support, as well as subjective and physical health and SES underscored the construct validity.

**Conclusion:**

Overall, findings underscore that resilient coping is a dynamic construct with considerable stability. The scale showed good psychometric properties including test-retest reliability over four months to two years. We found that it is not only important to describe the level of resilient coping, but also its stability.

## 1. Introduction

The Brief Resilience Coping Scale (BRCS) is a measure comprised of 4 items to assess the ability to cope with stress in an adaptive manner [1]. Given its brevity, it is highly suitable for epidemiological studies. In previous studies on the German general population, individuals with strong resilient coping reported reduced distress and somatoform symptoms, even when having sustained adverse childhood experiences [2, 3]. López-Pina et al. [4] suggested that the measure could identify patients with limited resilience, facilitating the initiation of suitable interventions. In the past, the one-dimensional scale demonstrated good internal consistency, test-retest reliability, psychometric robustness and sensitivity to cognitive-behavioural interventions [1, 5].

Regarding stressful events, results from studies performed during the pandemic in Italy, Australia and Thailand indicate that the BRCS is a useful tool to measure resilience during the pandemic [6–8]. It was also found that, during lockdown, the levels of resilient coping were significantly lower compared to normative data [9]. Results from 26 general population surveys link younger age, female sex, poorer financial situation and the presence of medical conditions with low levels of resilient coping during the COVID-19 pandemic [10]. Individuals with high levels of resilience showed higher emotional stability during lockdown and a reduction in perceived stress after lockdown restrictions had been lifted [11]. Lower levels of resilient coping have also repeatedly been found to be associated with higher levels of depression and anxiety after previous trauma exposure or a multiple sclerosis diagnosis [12–15]. Moreover, resilient coping was also found to have a direct effect on the distress and burnout of caregivers, physicians and teachers during the pandemic [16–19].

There is, however, a lack of published data regarding the BRCS’s test-retest reliability in a German sample during the pandemic and over extended periods. Data from the Gutenberg Health Study (GHS) and Gutenberg COVID Study (GCS), collected both before and during the pandemic, therefore offers a unique chance to study the BRCS’ longitudinal test-retest reliability and temporal stability. Based on this data, this paper aims to

- analyse changes in BRCS over the course of the pandemic,
- test the psychometric properties of the BRCS including test-retest reliability and measurement invariance,
- analyse sex, age group, relationship status, as well as poverty status as predictors of change,
- identify associations between the BRCS and variables such as psychosocial stress, depressiveness, anxiety, social support, and subjective mental and physical health in order to assess construct validity.

## 2. Methods

### 2.1. Data

We draw the data from our main sample from the Gutenberg COVID-19 Study (GCS). The GCS was initiated after the outbreak of SARS-CoV-2. Respondents who have already participated in the Gutenberg Health Study (GHS, [20]) prior to the beginning of the pandemic, were invited back in order to investigate the epidemiology of COVID-19 within the population. The participants were recruited in the target area of Mainz/Mainz-Bingen by drawing random samples from the resident’s registration office. The sample was stratified by sex, age, and place of residence.

The baseline examination of the GCS took place from October 2020 to April 2021 (T1). The first GCS follow-up was conducted from March 2021 to June 2021 (T2). Finally, the second follow-up occurred from May 2022 to November 2022 (T3). At the study center, participants underwent a computer-assisted personal interview and sampling of biomaterial. In preparation for their appointments, study participants were sent questionnaires in advance.

In total, the GCS sample comprises of *N* = 8121 individuals from the GHS and an additional *N* = 2129 newly recruited younger (aged 25–44) individuals for the GCS. For the present study, we included respondents with available data at all three GCS examination time points and complete and valid BRCS data. This left us with a sample of *N* = 6009 individuals. Sensitivity analysis was performed on a subsample of *N* = 3414 with valid BRCS data at all four time points, including additional pre-pandemic BRCS data from the 10-year follow-up of the GHS (2017–2020).

We attended the principles of Good Clinical Practice (GCP), Good Epidemiological Practice (GEP), and the ethical guidelines set forth in the Declaration of Helsinki throughout the entire process of the study design, execution, and analysis. We additionally adhered to the requirements outlines in the Federal Data Protection Act. Both the Ethics Committee of the Rhineland-Palatinate Medical Association and the Data Protection Officer of the Johannes Gutenberg-University Medical Center Mainz thoroughly assessed all pertinent documentation for both the Gutenberg Health Study and the Gutenberg COVID-19 Study, granting their approval. Furthermore, the data protection commissioner of Rhineland-Palatinate approved the selection of the sample through citizens’ registration offices.

### 2.2. Measures

#### 2.2.1. Brief resilient coping scale (BRCS)

Resilient coping was assessed using the four-item Brief Resilience Coping Scale (BRCS, [1, 5]). Respondents were asked to rate their ability to alter difficult situations, to adjust or control one’s reaction, and to find ways to make up for losses in life on a scale from *1* = does not apply at all to *5* = does apply fully. The sum score of the BRCS, therefore, ranges from 4, indicating the lowest, to 20, indicating the highest level of resilient coping.

#### 2.2.2. Psychological variables

In addition to the BRCS, we also collected data on depressive symptoms using the Patient Health Questionnaire (PHQ-9, [21, 22]), anxiety symptoms using the Generalized Anxiety Disorder Screener (GAD-2, [23–26]). Psychosocial stress was assessed using the PHQ stress module [27, 28], a 10-item subscale of the PHQ that assesses psychosocial stressors such as concerns about one’s health and weight, stress at work, worries about one’s financial situation, low sexual desire, among others on a 4-point scale. Loneliness using the UCLA Loneliness Scale [29–31], and social support using the Brief Social Support Scale (BS-6, [32]).

#### 2.2.3. Health related variables

Subjective physical and mental health were inquired using a single item each that asked the respondents to rate their health status on a 4-point scale ranging from *1* = very good to *4* = bad.

#### 2.2.4. Sociodemographic variables

Additionally, we inquired about age, sex, migration background, education, employment (no, irregular, part-time, fulltime current employment), partnership (yes/no) and socioeconomic status (SES) using the index of Lampert and Kroll [33].

Using the definition of relative poverty by the European Union Statistics on Income and Living Conditions (EU-SILC, [34]), we defined respondents at-risk-of-poverty if their net equalized income was under 60% of the median net equalized income of all households. Net equalized income was calculated by dividing the total monthly household income by a weighted household size: The first adult of a household contributed 1.0 unit to the household weight while every additional adult from an age of 14 increased the household weight by 0.5. Every child under the age of 14 increased the household weight by 0.3. The median net equalized income of 2019 was at 1790€, therefore, a net equalized income of <1074€ was identified to be the threshold of being at-risk-of-poverty.

### 2.3. Statistical procedure

We first took a look at the characteristics of our sample by calculating absolute and relative frequencies for categorical variables and means with standard deviations for continuous variables.

To analyze the changes in BRCS over the course of the pandemic, we estimated the means and standard deviations of the BRCS sum score and the individual items at all three measurement time points. We additionally expressed mean differences using Cohen’s *d* effect sizes and tested for their significance using t-tests.

To complete the next aim of our study and test the psychometric properties, we began by testing the internal stability of the sum score using Cronbach’s alpha (*α*) and McDonald’s total omega (*ω*_*t*_). The temporal stability of the sum score and the individual items was estimated using Pearson’s correlation coefficient. Discriminatory power coefficients were calculated using the corrected correlation between the individual item and the total score. In order to test factor validity, confirmatory factor analysis (CFA) determined if the pre-defined general factor model with all four items loading onto one factor was appropriate for the BRCS. We tested this for all three time points separately. Deviations from the normal distribution at all three pandemic time points were corrected using maximum likelihood estimation. This is a robust method in dealing with violations of normality [35]. The goodness-of-fit was determined using the following four criteria: the Comparative Fit Index (CFI), the Tucker Lewis Index (TLI), the Standardized Root Mean Squared Residual (SRMR), as well as the Root Mean Square Error of Approximation (RMSEA). A RMSEA and SRMR value of < 0.050 indicates a good fit while a value between 0.050 and 0.080 indicates a reasonable fit. For CFI and TLI, a value of > 0.950 demonstrates a good fit of a model [36, 37]. Subsequently, we tested for measurement invariance across the time points. We used the guidance of Meredith and Teresi’s [38] sequential strategy. Within this framework, we evaluated configural, weak, strong, and strict invariance by introducing more restrictions as the invariance models progress. Configural invariance assumes that there might be variations in the loadings, intercepts, and variances of the latent construct among the different groups or time points. Therefore, this model is completely unrestricted. Loadings are constrained to be equal across groups or time points if weak measurement invariance is assumed. To test for strong measurement invariance, we constrained both factor loadings and item intercepts to be equal across time points. Finally, for strict measurement invariance we assumed factor loadings, item intercepts and residual variances to be equal across time points. All invariance models were then compared to the more stringent model. The most commonly applied method for testing a model fit is the chi-square-test. However, due to its affectability by large sample sizes, which potentially leads to the false rejection of reasonable models, we relied on the abovementioned four fit indices to compare the model fits. Full invariance can be assumed if the CFI difference between all invariance models does not surpass the threshold of ΔCFI ≥ 0.010, as proposed by Chen [39–41].

Next, we looked at differences in changes in resilient coping between different sociodemographic groups by calculating the means of each group and testing the significance of their mean differences compared to the other group using t-tests. We also checked if the temporal stability differed across groups.

The fourth goal of our study was to further test for associations of the BRCS with relevant sociodemographic and psychological factors by using Pearson’s correlation coefficients as well as by testing for mean score differences between groups. Age groups (< 60 vs. ≥ 60) were formed in such a way that they approximately divide the sample in half.

Finally, we performed a sensitivity analysis and checked for significant pandemic-related differences by checking for significant changes in resilient coping since the start of the pandemic compared to a pre-pandemic measurement time point. We additionally assessed test-retest stability between pre-pandemic and pandemic time points using Pearson correlations. All analyses were conducted using R version 4.1.1 (packages: psych [42], lsr [43], lavaan [44], semTools [45]).

## 3. Results

### 3.1. Sample characteristics

Of the *N* = 10250 respondents at the GCS baseline examination (T1), *N* = 6009 provided valid and complete BRCS data for all three pandemic time points. Sociodemographic characteristics of the sample are shown in Table 1. The sample consisted of *N* = 3110 (51.8%) female and *N* = 2899 (48.2%) male respondents with a mean age of 56.57 (*SD* = 14.70).

**Table 1:** Sample Characteristics at T1, *N* = 6009.

	Available data	Sample	*α*	*ω*_*t*_
Sex (female, yes %)	6009	3110 (51.8%)		
Age (mean)	6009	56.57 (14.7)		
Age group (%)	6009			
25–34		515 (8.6%)		
35–44		914 (15.2%)		
45–54		1121 (18.7%)		
55–64		1467 (24.4%)		
65–74		1257 (20.9%)		
75+		735 (12.2%)		
Migration background (yes, %)	6004	1186 (19.8%)		
Partnership (yes, %)	4543	4113 (90.5%)		
SES (mean)	5766	14.96 (4.07)		
Employment (%)	5679			
no current employment		1868 (32.9%)		
irregularly		325 (5.7%)		
part-time		1057 (18.6%)		
fulltime		2429 (42.8%)		
Education (%)	4936			
General secondary school degree		1030 (20.9%)		
Secondary school degree		1303 (26.4%)		
Academic school degree		2588 (52.4%)		
Other degree		3 (0.1%)		
No degree		12 (0.2%)		
Equalized income (mean)	5680	3024.69 (1903.88)		
At-risk-of-poverty (yes, %)	5680	196 (3.4%)		
Living environment	6009			
urban (Mainz)		2742 (45.6%)		
rural (Bingen)		3073 (51.2%)		
other		194 (3.2%)		
Subjective physical condition (mean)	6007	1.96 (0.55)		
Subjective mental condition (mean)	6007	2.02 (0.60)		
Diabetes (yes, %)	5997	484 (8.1%)		
Obesity (yes, %)	6006	3590 (59.8%)		
Hypertension (yes, %)	5993	3023 (50.4%)		
CVD (yes, %)	5993	766 (12.8%)		
COPD (yes, %)	6002	373 (6.2%)		
Cancer (yes, %)	5998	855 (14.3%)		
History of depression (yes, %)	5993	728 (12.1%)		
History of Anxiety (yes, %)	5991	412 (6.9%)		
PHQ-Stress (mean)	5059	4.05 (3.17)	0.73	0.76
PHQ-9 (mean)	5770	4.27 (3.73)	0.83	0.86
GAD-2 (mean)	4714	0.81 (1.11)	0.75	0.75
UCLA loneliness (mean)	5679	3.64 (2.38)	0.76	0.78
BS-6 (mean)	5767	20.83 (3.71)	0.89	0.94
BRCS (mean)	6009	14.77 (3.11)	0.76	0.79

*Note*. CVD–cardio vascular diseases; COPD–chronic obstructive pulmonary disease; PHQ–Patient Health Questionnaire; GAD-2 –Generalized Anxiety Disorder screener; BS-6 –Brief Social Support Scale; BRCS–Brief Resilient Coping Scale.

### 3.2. BRCS mean scores and item characteristics

The BRCS score yielded good reliability at all three examination time points with Cronbach’s alpha ranging from 0.76 to 0.78 and McDonald’s omega ranging from 0.79 to 0.81, as can be seen in Table 2. The mean score of the sample decreased slightly from 14.77 (*SD* = 3.11) to 14.45 (*SD* = 3.25) between the first and second measurement time point and subsequently increased again at the third time point (*M* = 14.82, *SD* = 3.19). These differences had very low effect sizes of *d* = 0.10 between T1 and T2, *d* = 0.11 between T2 and T3, and *d* = 0.01 between T1 and T3, respectively. However, the differences between the time points were statistically significant (*p* < 0.001) with the exception of the difference between T1 and T3.

**Table 2:** BRCS item and score characteristics.

Item / score	T1	T2	T3	Δ_t2-t1_	Δ_t3-t2_	Δ_t3-t1_	ES_t2-t1_	ES_t3-t2_	ES_t3-t1_	r_it t1_	r_it t2_	r_it t3_	r_tt t2-t1_	r_tt t3-t2_	r_tt t3-t1_
	*M (SD)*	*M (SD)*	*M (SD)*	*M (SD)*	*M (SD)*	*M (SD)*									
BRCS sum score	14.77 (3.11)	14.45 (3.25)	14.82 (3.19)	-0.33*** (2.89)	0.36*** (3.07)	0.03 (3.06)	0.10	0.11	0.01	*α* = 0.76 *ω* = 0.79	*α* = 0.79 *ω* = 0.81	*α* = 0.78 *ω* = 0.80	0.589	0.539	0.527
I look for creative ways to alter difficult situations.	3.83 (1.02)	3.71 (1.07)	3.84 (1.05)	-0.13*** (1.05)	0.14*** (1.11)	0.01 (1.11)	0.12	0.13	0.01	0.57	0.61	0.59	0.496	0.454	0.425
Regardless of what happens to me, I believe I can control my reaction to it.	3.68 (0.87)	3.62 (0.92)	3.72 (0.88)	-0.07*** (0.92)	0.10*** (0.97)	0.04* (0.95)	0.07	0.12	0.04	0.44	0.49	0.46	0.472	0.414	0.407
I believe I can grow in positive ways by dealing with difficult situations.	3.82 (0.97)	3.74 (0.99)	3.81 (0.98)	-0.08*** (0.99)	0.07** (1.01)	-0.02 (1.01)	0.08	0.07	0.01	0.65	0.68	0.68	0.490	0.470	0.462
I actively look for ways to replace the losses I encounter in life.	3.44 (1.20)	3.39 (1.18)	3.44 (1.18)	-0.05* (1.21)	0.05* (1.21)	-0.00 (1.26)	0.04	0.04	0.00	0.56	0.62	0.62	0.489	0.470	0.443

*Note*. Δ = difference between the time points; ES = effect size of difference of the scores between the time points measured by Cohen’s *d*; r_it_ = discriminatory power calculated by corrected item-total correlations; r_tt_ = Pearson correlation coefficient for test-retest testing; α = Cronbach’s Alpha; ω = McDonald’s Omega; *** p < 0.001, ** p < 0.01, * p < 0.05.

All items contributed to the decrease between T1 and T2 as well as to the increase between T2 and T3 with effect sizes ranging from *d* = 0.04 (looking for ways to replace the losses in life) to *d* = 0.13 (looking for creative ways to alter difficult situations). Additionally, all items contributed to the BRCS total score at all three time points with discrimination power coefficients ranging from 0.44 to 0.68.

The test-retest correlations of the total BRCS score were 0.589 between T1 and T2, 0.539 between T2 and T3, and 0.527 between T1 and T3, while the test-retest correlations of the single items ranged from 0.407 to 0.496.

### 3.3. Measurement invariance

The results of the confirmatory factor analysis (CFA) and measurement testing are presented in Table 3. When analyzed separately, the BRCS data of all three time points yielded good model fits. The CFI, in particular, indicated a very good fit (T1: 0.988, T2: 0.991, T3: 0.990). We saw that the fits of the model did not change significantly by introducing further restraints. Furthermore, the difference in CFI did not exceed the threshold of a change of more than 0.01, indicating that full measurement invariance was able to be established.

**Table 3:** Confirmatory factor analysis and measurement invariance.

	CFI	TLI	RMSEA	SRMR	Δ CFI	Δ TLI	Δ RMSEA	Δ SRMR
T1	0.997	0.991	0.035	0.020				
T2	0.998	0.995	0.030	0.017				
T3	0.998	0.995	0.030	0.018				
Configural	0.997	0.992	0.035	0.017				
Metric	0.997	0.995	0.027	0.017	0.000	0.003	-0.008	0.000
Scalar	0.996	0.996	0.027	0.019	-0.001	0.001	0.000	0.002
Strict	0.995	0.996	0.025	0.023	-0.001	0.001	-0.002	0.004

*Note*. CFI = Robust Comparative Fit Index; TLI = Robust Tucker-Lewis Index; RMSEA = Robust Root Mean Square of Approximation; SRMR = Standardized Root Mean Square Residual; Δ = difference in model fits between sequential models.

### 3.4. Associations with sociodemographic and other psychological factors

In Table 4 we compared the means, as well as changes in BRCS between different sociodemographic groups. We found significant mean differences for all time points between people over and under 60. There were also significant mean differences between men and women as well as for being at-risk-of-poverty and not, but only for the first two time-points. We did not find any significant mean difference between people with a partner and single people. In regards to changes in score between the time points T1 and T2, we found a significant difference only for respondents under the age of 60, whose score decreased significantly less between T1 and T2. For the changes in score between T2 and T3, we observed that the score of women and people under the age of 60 increased significantly more than those of men and people over the age of 60. We also found that the temporal stability in BRCS between men and women differs: The temporal stability amongst women was higher than among men (Women T1-T2: 0.601 vs. Men T1-T2: 0.574; Women T2-T3: 0.553 vs. Men T2-T3: 0.524). There were also differences between age groups with the temporal stability among the older group being lower across both tests. For partnership and poverty status, we observed differences for the T1-T2 test-retest only with the group of people with a partner and the group of people at-risk-of-poverty demonstrating higher levels of temporal stability.

**Table 4:** Changes in BRCS sum score by sex, age group, partnership, and being at-risk-of-poverty.

				Change T1-T2				Change T2-T3					
	T1	T2	T3	Δ T2-T1	decrease (%)	no change (%)	increase (%)	Δ T3-T2	decrease (%)	no change (%)	increase (%)	r_tt t12-t1_	r_tt t13-t2_
Women	14.65 (3.16)	14.33 (3.29)	14.80 (3.18)	-0.33 (2.88)	1317 (43.7%)	673 (22.3%)	1027 (34.0%)	0.46 (3.04)	984 (33.0%)	578 (19.4%)	1420 (47.6%)	0.601	0.553
Men	14.90 (3.06)	14.58 (3.20)	14.83 (3.20)	-0.33 (2.90)	1244 (44.2%)	621 (22.1%)	949 (33.7%)	0.25 (3.11)	992 (35.5%)	593 (21.3%)	1206 (43.2%)	0.574	0.524
*Group difference (p-value)*	***0*.*002***	***0*.*004***	*0*.*756*	*0*.*981*				***0*.*007***					
< 60	14.91 (2.87)	14.65 (2.98)	15.14 (2.86)	-0.26 (2.49)	1439 (43.1%)	781 (23.4%)	1118 (33.5%)	0.47 (2.73)	1049 (31.6%)	695 (21.0%)	1573 (47.4%)	0.638	0.562
> 60	14.58 (3.40)	14.19 (3.56)	14.38 (3.54)	-0.44 (3.34)	1122 (45.0%)	513 (20.6%)	858 (34.4%)	0.20 (3.48)	927 (37.7%)	476 (19.4%)	1053 (42.9%)	0.539	0.512
*Group difference (p-value)*	***0*.*000***	***0*.*000***	***0*.*000***	***0*.*017***				***0*.*001***					
with partner	14.76 (3.17)	14.42 (3.32)	14.75 (3.30)	-0.37 (3.02)	1761 (44.1%)	885 (22.2%)	1348 (33.7%)	0.33 (3.16)	1384 (35.0%)	791 (20.0%)	1776 (45.0%)	0.569	0.540
without partner	14.63 (3.39)	14.24 (3.59)	14.54 (3.44)	-0.44 (2.95)	185 (45.0%)	84 (20.4%)	142 (34.6%)	0.30 (3.40)	139 (34.1%)	74 (18.2%)	194 (47.7%)	0.642	0.537
*Group difference (p-value)*	*0*.*425*	*0*.*317*	*0*.*221*	*0*.*637*				*0*.*859*					
At-risk of poverty	13.89 (3.61)	13.96 (3.40)	14.38 (3.48)	0.00 (3.07)	86 (45.3%)	35 (18.4%)	69 (36.3%)	0.43 (3.31)	60 (31.9%)	44 (23.4%)	84 (44.7%)	0.612	0.536
Not at-risk	14.82 (3.10)	14.49 (3.23)	14.84 (3.18)	-0.34 (2.88)	2336 (43.9%)	1189 (22.3%)	1800 (33.8%)	0.34 (3.07)	1818 (34.5%)	1070 (20.3%)	2384 (45.2%)	0.588	0.538
*Group difference (p-value)*	***0*.*000***	***0*.*025***	*0*.*051*	*0*.*110*				*0*.*696*					

*Note*. r_tt_ = Pearson correlation coefficient for test-retest testing.

Subsequently, we evaluated the associations between the BRCS sum score at all three measurement time points with relevant sociodemographic and psychological factors (see Table 5). Depressiveness, anxiety, psychosocial stress, loneliness, age, subjective mental and physical health were found to have significant negative associations with the BRCS across all three time points with Pearson’s correlation coefficients ranging from -0.084 (PHQ stress) to -0.204 (subjective mental health). Social support and SES consistently demonstrated a significant positive association with the BRCS; Pearson’s correlation coefficients ranged from 0.153 (BS-6) to 0.207 (SES). Being female and being at-risk-of-poverty were found to have smaller significant negative associations with the BRCS at the first two measurement time points, but not at the third. Having a partner or a migration background was not associated with the BRCS at any time point.

**Table 5:** Pearson correlation of the BRCS sum score at all pandemic time points with relevant factors.

	T1	T2	T3
PHQ-9	-0.195***	-0.168***	-0.127***
GAD2	-0.181***	-0.167***	-0.116***
PHQ-Stress	-0.154***	-0.141***	-0.084***
BS-6	0.185***	0.153***	0.155***
UCLA loneliness scale	-0.166***	-0.156***	-0.114***
Age (continuous)	-0.057***	-0.080***	-0.134***
Female Sex	-0.040**	-0.038**	-0.004
Partnership	0.012	0.015	0.018
At-risk-of-poverty	-0.054***	-0.030*	-0.026
SES	0.207***	0.195***	0.184***
Migration background	0.011	-0.015	0.002
Subjective physical health	-0.150***	-0.140***	-0.157***
Subjective mental health	-0.204***	-0.177***	-0.143***

*Note*.

*** p < 0.001

** p < 0.01

* p < 0.05. PHQ–Patient Health Questionnaire; GAD-2 –Generalized Anxiety Disorder screener; BS-6 –Brief Social Support Scale; BRCS–Brief Resilient Coping Scale; SES–socioeconomic status. Note that the factors correlated with the BRCS sum score were always collected at the same measurement time point as the BRCS sum score.

### 3.5. Sensitivity analysis

As a next step, we tested the score’s sensitivity to stressful events such as the onset of a pandemic. For this, we drew additional data from a pre-pandemic measurement time point and tested the mean difference as well as performed a test-retest correlation with the three pandemic time points. The results are shown in Table 6. Compared to the pre-pandemic time point, which yielded a mean score of 15.46 (*SD* = 3.03), we found significant (p < 0.001) decreases in BRCS at all three pandemic time points with means ranging from 14.48 (*SD* = 3.31) to 14.80 (*SD* = 3.17). The testing for test-retest reliability yielded good results with correlation coefficients ranging from 0.471 to 0.528.

**Table 6:** Sensitivity analysis of the BRCS between pre-pandemic and pandemic time points (*N* = 3414).

	T0	T1	T2	T3
BRCS mean (SD)	15.46 (3.03)	14.80 (3.17)	14.48 (3.31)	14.73 (3.32)
Mean difference test (compared to T0)	-	0.000	0.000	0.000
*r*_*tt*_	-	0.513	0.528	0.471

*Note*. *r*_*tt*_ = Pearson correlation coefficient to test for test-retest reliability.

## 4. Discussion

The COVID-19 pandemic and the measures put in place to contain its spread have comprised novel mental and physical health threats, pervasive societal and daily-life restrictions, as well as changes of social and work life on an unprecedented, world-wide basis over an extended period of time. This allows us to gain unique insights into the role of resilience. As a first aim of this study, we, therefore, analyzed the changes of resilient coping over the three pandemic time points. We found a significant decrease between the first and the second time points and a significant increase to the third time point. However, effect sizes of these changes were small (*d* = 0.10 between T1 and T2, *d* = 0.11 between T2 and T3). Compared to data from BRCS validation papers, the means of our data (T1: 14.77 (*SD* = 3.11), T2: 14.45 (*SD* = 3.25), T3: 14.82 (*SD* = 3.19) corresponded to other community samples, with Sinclair and Wallston [1] reporting an overall mean of 14.81 (*SD* = 2.95) and Kocalevent et al. [5] reporting a mean of 14.9 (*SD* = 3.3) for men and a mean of 14.6 (*SD* = 3.1) for women. We do, however, find a dip in the BRCS score at the second measurement point. This may be explained by the tightening of COVID restrictions in April of 2021 when the German government invoked an emergency brake by reducing social contacts and by putting a nighttime curfew into place. This happened following a prior loosening of lockdown measures earlier in the year which was highly controversial [46]. Similarly, Gullo et al. [9] reported decreases in BRCS during lockdown in the first half of 2020 compared to normative data. Following this dip of resilient coping, scores went up to the previous levels in 2022.

As the next step we tested the psychometric properties of the BRCS. The scale was found to have a good internal consistency with Cronbach’s alpha ranging from 0.76 to 0.78 and McDonald’s omega ranging from 0.79 to 0.81. These values are slightly higher than those reported by Sinclair and Wallston [1] as well as Kocalevent et al. [5], whose alphas ranged from 0.64 to 0.76. The test-retest correlation coefficients of our study ranged from 0.527 to 0.589, which are lower than the values reported by Sinclair and Wallston [14] (*r* = 0.71 after 5 weeks and *r* = 0.68 after three months), but are similar to those of another study of a German sample during the same time period with *r* = 0.60 [47]. As more time elapsed in our study (from four months to two years), consistent with previous studies we would presume that temporal stability decreases as time between measurement points increases [48, 49]. We additionally tested the measurement invariance across all three pandemic time points, which–to our knowledge–has not yet been done with the BRCS, and were able to establish full invariance. This indicates that the instrument is being interpreted the same way at all three time points, allowing for a fair comparison of the means over time.

When we associated the BRCS differences with sex, age group (< 60 vs. ≥ 60), partnership status (with vs. without a partner), as well as poverty (being vs. not being at-risk-of-poverty), we found significant mean differences for all time points between people over and under 60 with higher resilient coping in the younger age group. Significant effects were found for sex and risk-of-poverty for the first two time points. Corresponding to Wong et al. [10], female sex and risk-of-poverty were associated with lower resilient coping. In Wong et al.’s study, however, it was found that younger age was associated with lower levels of resilient coping, which contradicts our findings. This might be due to the fact that our sample is relatively old with a mean age of 56.6 (*SD* = 14.7) years; younger people, who were more affected by distress (and a negative impact to their resilient coping skills) during the pandemic [50, 51], were underrepresented in our sample. If our sample had included more younger people with symptoms of distress, we might have come to different results.

The temporal stability among women and participants under the age of 60 was higher than among men and people over 60 years. Furthermore, we found higher temporal stability (T1-T2 test-retest coefficients) for participants with (vs. without) a partner and those at-risk-of-poverty. The lowest temporal stability, however, was found for men (*r*_*tt t3-t2*_ = 0.524). These findings show that not only is it important to describe the level of resilient coping, but also its stability. For example, high stability in those at-risk-of-poverty could mean that it is hard for economically disadvantaged to sustain resilient coping under stressful conditions. Previously, we were able to find that those at-risk-of-poverty indeed suffered more COVID-19 related economic disadvantages compared to those who were better off [52]. High stability during stressful times might make it harder for these population groups to ‘bounce back’ after major economic setbacks. On the other hand, a partnership may also enhance stability of resilient coping, regardless of its level.

As we had expected, depressiveness, anxiety, psychosocial stress, loneliness, subjective mental and physical health had significant negative associations with the BRCS across all three time points, while social support and SES consistently demonstrated a significant positive association with the BRCS. These findings indicate construct validity and are in line with results from previous studies [10, 12–19].

Subgroup analysis confirmed significantly lower BRCS levels (means ranged from 14.48 to 14.80) during all three pandemic time points compared to a pre-pandemic measurement point (*M* = 15.46, *SD* = 3.03). This finding is in accordance with Gullo et al. [9], who found BRCS levels during the pandemic to be lower compared to (non-pandemic) normative data. This finding indicates that the BRCS in the general is sensitive to large-scale external stressors. Though, it must be noted that our pre-pandemic data yields signficantly higher BRCS mean scores than those reported in previous validation papers. Our BRCS score from our pandemic data, however, as mentioned above, were more aligned with those reported in these papers. This raises the question if the GHS and GCS samples are generally more resilient than other samples.

### 4.1. Limitations

While we have a unique data base over an extended time period before and during the pandemic, the community sample was relatively old with a mean age of 56.6 years and financially better off with only 3.4% people being at-risk-of-poverty compared to the 14.7% reported by the German Federal Statistical Office [53], which limits the representativeness of our results and, therefore, the mean scores should be interpreted with caution.

Additionally, even though 10250 people participated at the GCS baseline examination (T1), only *N* = 6009 participated in all three measurements with valid data. This raises the question of who drops out of a longitudinally study. It cannot be precluded that unwell people with low resilience scores become unfit for these types of examinations, which might also explain why our BRCS score are so high compared to normative data. While effect sizes of changes of resilient coping were small, these may still be meaningful in the general population as we could show that resilience varies with sociodemographic characteristics. Finally, some of the study took place during the roll-out of vaccinations. The access to vaccines and the subsequent loosening of lockdown measures might have had an effect on the BRCS.

## 5. Conclusion

Resilience is a dynamic construct. Indeed, the BRCS demonstrated a good temporal stability and proved to be a reliable instrument for longitudinal studies. Furthermore, we found that the score is sufficiently sensitive to external stressors such as a global pandemic, rendering it to be a valuable instrument for implementation in future studies on resilient coping. Finally, sociodemographic factors such as sex, age, and poverty were significantly associated with changes in BRCS, therefore providing more insight on factors influencing changes over time.
